# Local Deformation and Texture of Cold-Rolled AA6061 Aluminum Alloy

**DOI:** 10.3390/ma11101866

**Published:** 2018-10-01

**Authors:** Diaoyu Zhou, Wenwen Du, Xiyu Wen, Junwei Qiao, Wei Liang, Fuqian Yang

**Affiliations:** 1 College of Materials Science and Engineering, Taiyuan University of Technology, Taiyuan 030024, China; Diaoyu.Zhou@uky.edu (D.Z.); qiaojunwei@gmail.com (J.Q.); 2 Materials Program, Department of Chemical and Materials Engineering, University of Kentucky, Lexington, KY 40506, USA; xwen@secat.net; 3 Department of Science and Mathematics, Glenville State College, Glenville, WV 26351, USA; wenwen.du@gmail.com

**Keywords:** cold rolling, aluminum alloy, microstructures, textures, microindentation

## Abstract

Using cold rolling, we plastically deform AA6061 sheets at room temperature and investigate the variations of the microstructures, textures and local deformation of the cold-rolled AA6061 sheets as functions of thickness reduction (Δt/t_0_, t_0_ and t are the thicknesses of the AA6061 sheet before and after the cold rolling, respectively). The volume fraction of total deformation texture is relatively independent of the thickness reduction for Δt/t_0_ ≤ 30%, and becomes an approximately linearly increasing function of the thickness reduction for Δt/t_0_ > 30%. Increasing the thickness reduction causes the increase of the Vickers hardness of the cross-section of the cold-rolled sheets, which exhibits a similar increase trend to the volume fraction of total deformation texture for Δt/t_0_ > 30%. A simple relation between the Vickers hardness and the thickness reduction is established and is used to curve-fit the experimental results.

## 1. Introduction

The demand for producing light vehicles with reduced fuel consumption has led to an increasing interest in the applications of AA6xxx aluminum (Al) alloy instead of steel for automotive industry due to reasonable strength, good formability, and corrosion resistance of AA6xxx Al alloy [1,2,3,4,5]. However, the ridging and roping phenomena, which have been widely observed in some AA6xxx Al alloy after being stretched along the transverse direction (TD) [6,7,8], has imposed a great challenge to the applications of AA6xxx Al alloy, since surface appearance is one of the important considerations for the applications of sheet metals in the automobile industry. 

It is known that the texture, a spatial distribution of specific crystallographic orientations, plays an important role in the appearance of ridging or roping in sheet metals [8,9,10], and rolling of Al alloys can introduce mechanical anisotropy of Al alloy sheets through the changes of crystallographic texture. Using cold rolling and accumulative roll bonding (ARB) at room temperature, Kashihara et al. [11] investigated the ARB-induced variations of microstructure and textures in aluminum single crystals with {112} <111> crystallographic orientation, and found that the shear deformation caused the development of the (001) [110] orientation in the lower surface layer. Using X-ray diffraction and transmission electron microscopy, Toroghinejad et al. [12] analyzed the ARB-induced evolution of texture and microstructure in an AA5083 Al alloy, and noted the major texture components of Copper, Dillamore, Goss, and Brass in the ARB-pressed AA5083 Al alloy. Shabadi et al. [13] studied the effect of crystallographic texture on tensile behavior of the sheets of AA7020 Al alloy processed to different thicknesses, and observed the dependence of plastic flow and ductility on the texture. Yu et al. [14] observed that subsequent asymmetric cryorolling and ageing improved the mechanical properties of AA6061 sheets due to the grain refinement and precipitates of high density. Using spherical indenters, Yang et al. [15] investigated the local deformation behavior of cold-rolled AA6061 Al alloy with the indentation being performed on the rolling surface without analyzing the evolution of textures introduced by cold rolling. Currently, there are few studies focusing on the connection between the evolution of deformation-induced changes of textures and local mechanical behavior. 

AA6061 Al alloy is a precipitation-hardenable Al-Mg-Si alloy, with unique mechanical properties, including medium strength, formability, and corrosion resistance in comparison with other Al alloys [16,17], and has potential in the applications of automotive industry. This work is focused on the evolution of microstructures and textures of cold-rolled AA6061 Al sheets and their effect on the local mechanical deformation. In contrast to the work by Yang et al. [15], the indentation is performed on the transverse cross-section of the cold-rolled AA6061 Al sheets. The dependence of the energy dissipation on the thickness reduction is also analyzed.

## 2. Materials and Methods

The AA6061 Al sheets of 300 mm × 300 mm × 6.35 mm were purchased from Alfa Aesar, Ward Hill, MA, USA. Table 1 lists the composition of the AA6061 Al sheets. An AA6061 Al sheet was sliced into sheets of 5 mm × 10 mm × 6.35 mm via a band saw. Prior to the cold rolling, we annealed the sheets at 320 °C for 2 h in a furnace in air, and let the sheets cool down to room temperature in air in the furnace. The heat-treated sheets were rolled at room temperature on a rolling machine (CX210, Stanat MFG. Co. L.I.C, New York, NY, USA). During the rolling process, 10% thickness reduction (Δt = 0.635 mm, Δt/t_0_ = 10%, t_0_ and t are the thicknesses of the AA6061 sheet before and after the cold rolling) was achieved per rolling pass. Five different thickness reductions of 10%, 30%, 50%, 70%, and 90% were achieved. It took nine rolling passes to reach 90% thickness reduction. The resultant thickness reductions are 10%, 30%, 50%, 70%, and 90%, corresponding to the thicknesses of 5.72, 4.45, 3.18, 1.91 and 0.64, respectively. 

The as-annealed and cold-rolled samples were cut to obtain the specimens with the surface being parallel to the plane consisting of rolling direction and normal direction for the analyses of microstructures and the indentation tests. The surfaces of the specimens were manually ground and polished to obtain mirror-like surface. For the optical microscopy analysis, the mirror-like surface was electrochemically etched in a solution of 50 mL HBF_4_ and 950 mL DI water (deionized water) at a DC voltage of 18 V for 60 s. After the etching, the grain structures of the specimens were analyzed on an optical microscope (BX53M, Olympus Corp., Tokyo, Japan) under polarized light. A scanning electron microscope (SEM) (JEOL 5900 LV) was used to observe the particle structures in the specimens. 

The texture measurement of all the specimens was performed on the rolling surface, which was mechanically polished to reduce surface residual stresses and limit the stress effect on X-ray diffraction. The {111}, {200}, {220} and {311} pole figures were measured by the Schulz back-reflection method using CuK_α_ radiation for each specimen. The alpha rotation angle was in a range of 15–90° with the angle increment of 5°. The Tex Tools software (ResMat Corporation, Barcelona, Spain) was used to calculate the 3D ODFs (orientation distribution functions) and the volume fractions of texture components. A triple of Euler angles (φ_1_, Φ, φ_2_) was used to represent the orientation intensity, following Bunge’s notation. 

The local deformation behavior of the polished surface (transverse cross-section) of the cold-rolled specimens was measured on a Microhardness Tester (Micro Photonics, Irvine, CA, USA). The maximum normal load was 200 mN. The unloading time was the same as the loading time, and both were 30 s. There was no intermediate pause between the loading stage and the unloading stage. After unloading, the diagonal lengths of the indentation mark were measured on an optical microscope, and used to calculate the Vickers hardness. The area enclosed in the loading–unloading curve and the displacement axis was used to calculate the energy dissipation. 

## 3. Results and Discussion

**Figure 1:**
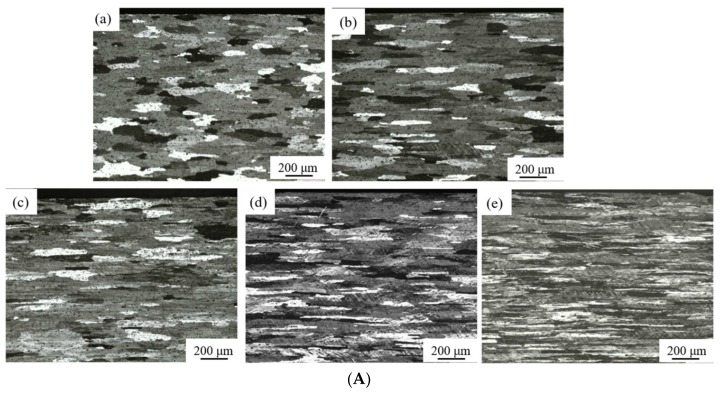

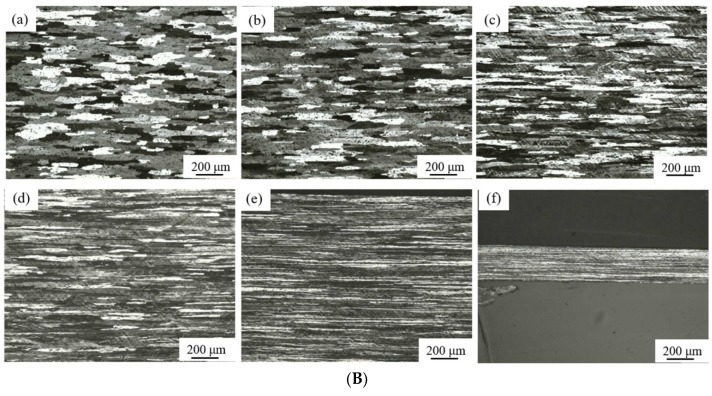
Optical metallographs of the as-annealed and cold-rolled AA6061 alloy; (**A**) surface region, and (**B**) center portion; (**a**) as-annealed, (**b**) Δt/t_0_ = 10%, (**c**) Δt/t_0_ = 30%, (**d**) Δt/t_0_ = 50%, (**e**) Δt/t_0_ = 70%, and (**f**) Δt/t_0_ = 90%.

Figure 1 shows optical metallographs of the surface and center portion of the as-annealed and cold-rolled AA6061 specimens. The as-annealed specimen has the largest average grain size, and the grains are presented in non-equiaxed shape. The non-equiaxed shape of the grains in the as-annealed specimen suggests that the AA6061 Al sheets received from Alfa Aesar had experienced plastic deformation, and there exist residual stresses in the AA6061 Al sheets. The annealing at 320 °C has caused the release of the residual stresses and the growth of grains accordingly. It is interesting to note that the grains near the surface of the as-annealed specimen have larger average size than those in the center portion of the as-annealed specimen. Such behavior reveals that there exists a difference of the grain growth between the material near the free surface and that in the center portion. The material near the free surface can relax the residual stresses at a higher rate than that in the center portion, which accelerates the grain growth near the free surface. 

According to Figure 1, the grains in the materials both near the free surface and around the center portion experienced elongation during the cold rolling, as expected, along the rolling direction. Increasing the thickness reduction increases the grain elongation along the rolling direction likely due to the compression deformation of grains during the rolling. There exists a slight difference in the grain sizes between the material near the free surface and the material around the center portion for a small reduction in the thickness. There are two possible mechanisms contributing this difference; one is the difference in the initial grain sizes, and the other is due to the difference in the strain state. As discussed above, the grains near the surface of the as-annealed specimen have larger average size than those in the center portion of the as-annealed specimen, which can produce grains of different sizes during the cold rolling. In addition, the interaction between the roller and the specimen during the rolling introduces larger shear strain in the material near the surface than that in the center portion, which likely produces different shape changes in grains. For the thickness reduction larger than 30%, there is no observable difference of the grain shapes between the region near the surface and that in the center portion.

**Figure 2:**
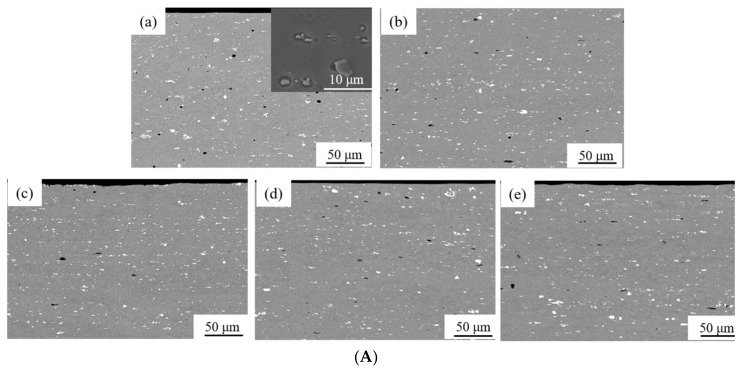

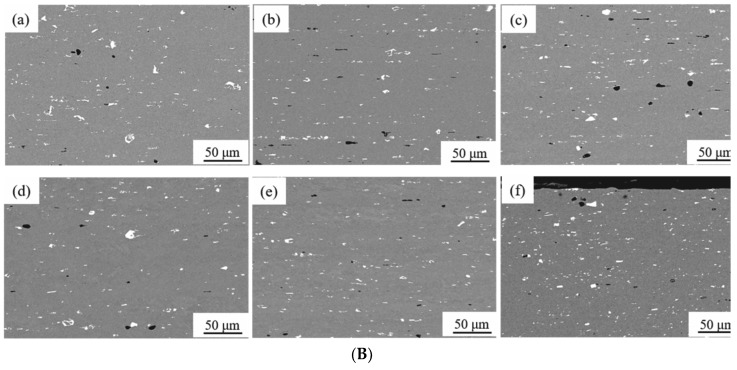
SEM images of the as-annealed and cold-rolled AA6061 alloy showing the dark and bright particles; (**A**) surface region, and (**B**) center portion; (**a**) as-annealed, (**b**) Δt/t_0_ = 10%, (**c**) Δt/t_0_ = 30%, (**d**) Δt/t_0_ = 50%, (**e**) Δt/t_0_ = 70%, and (**f**) Δt/t_0_ = 90%.

Figure 2 shows SEM images, in which particles (the second phase) are observed in the as-annealed and cold-rolled AA6061 alloy. More and smaller particles are presented near the surface than those around the center, which is likely due to the fast cooling during the manufacturing of the AA6061 Al sheet. The sizes of coarse particles are in a range of 3 to 10 µm, and the sizes of fine particles are less than 1 µm (as shown in the inset of Figure 2Aa). The particles are intermetallic compounds; one consists of Mg and Si (dark particles, Mg_2_Si), and the other consists of Al, Fe and Si (bright particles, Al(Fe,Mn)Si). 

### 3.1. Texture Analysis

**Figure 3:**
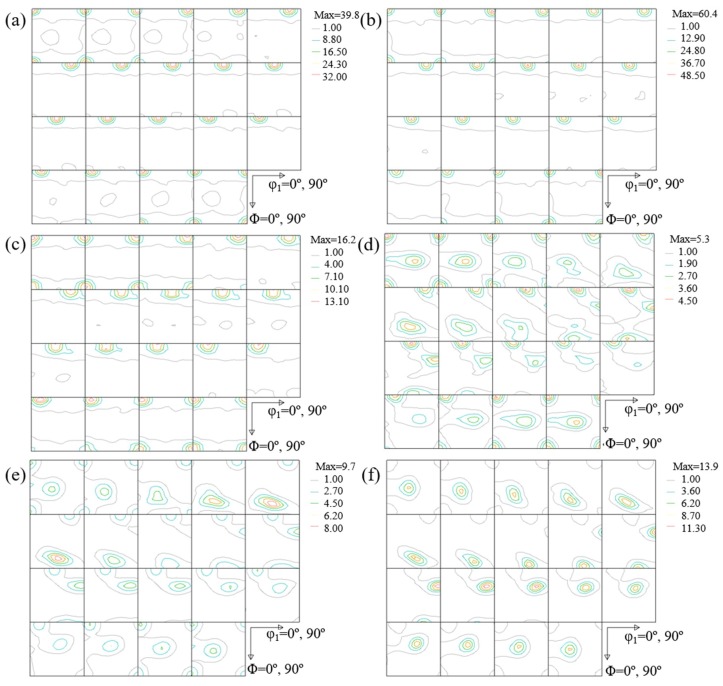
Three-dimensional ODF of the as-annealed and cold-rolled specimens; (**a**) as-annealed; (**b**) Δt/t_0_ = 10%; (**c**) Δt/t_0_ = 30%; (**d**) Δt/t_0_ = 50%; (**e**) Δt/t_0_ = 70%; and (**f**) Δt/t_0_ = 90%.

Figure 3 shows three-dimensional ODF of the as-annealed and cold-rolled specimens with different thickness reductions. It is evident that the texture of the AA6061 specimens mainly consists of two types of textures: recrystallization (softening) texture and deformation texture, which include six components of Cube ({100} <001>) (C), Goss ({011} <001>) (G), Brass ({011} <211>) (B), S ({123} <634>), Copper ({112} <111>), and R-cube ({100} <011>) (R-C) components. In general, Cube, Goss and R-cube components are referred to softening texture because of the negative rate of work hardening. Copper, S and Brass components are hardening orientations because the rate of work hardening is positive for deformed metal [10]. For the as-annealed specimen, there is a typical cube recrystallization texture of {100} <001>, suggesting that the heat treatment of the AA 6061 alloy at 320 °C accelerated the cube-oriented grains in the deformed matrix to undergo recovery in comparison with grains of other orientations [18]. 

The cold-rolled specimens exhibit the β-fiber rolling texture, which extends from the {112} <111> Copper component through the {123} <634> S component to the {011} <211> Brass component. Such a result is in accordance with the results given by Wen et al. [19]. There is almost no Cube component in the cold-rolled specimens. This trend suggests the transform of the Cube component to the components of Brass, Copper, and S. Increasing the thickness reduction causes the decrease of the intensity of the recrystallization texture, the increase of the intensity of the deformation texture, and the increase of the strength of the β-fiber rolling texture. The larger the thickness reduction, the stronger is the β-fiber rolling texture. The general features of the textures of the cold-rolled AA6061 alloy remain relatively unchanged, while the intensities of the textures increase with increasing the thickness reduction, as expected.

**Figure 4:**
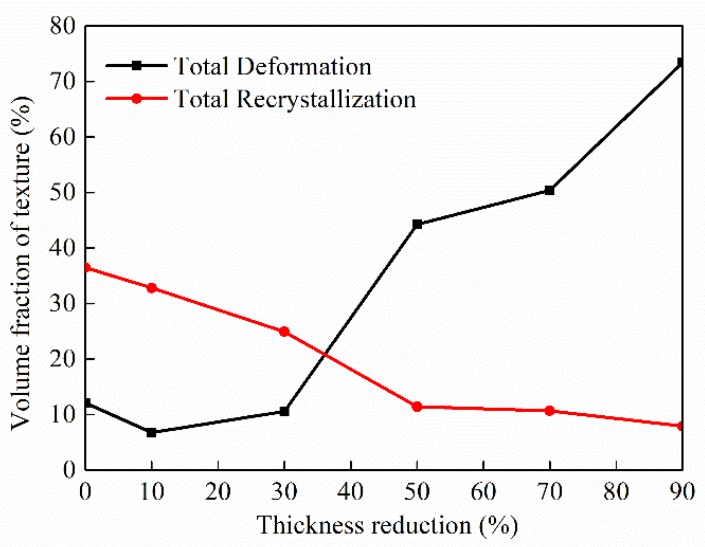
Variation of the volume fractions of textures with the thickness reduction.

The texture effect on the deformation of metals is dependent on the types of textures (recrystallization or deformation). It is of great importance to examine the variation of the total components of both the recrystallization texture (softening orientations) and the deformation texture (hardening orientations). Figure 4 shows the dependence of the volume fractions of the recrystallization texture and the deformation texture on the thickness reduction. It is evident that increasing the thickness reduction (plastic deformation) during the cold rolling results in the decrease of the volume fraction of the recrystallization texture and the increase of the volume fraction of the deformation texture, which can be attributed to the transform of the Cube component and random texture to the components of the deformation texture. 

### 3.2. Indentation Deformation

**Figure 5:**
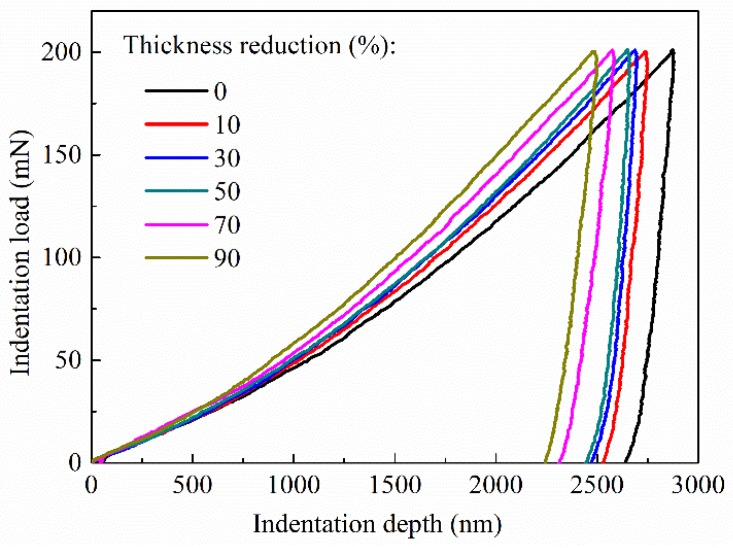
Load–displacement curves for indentations of the as-annealed and cold-rolled AA6061 alloy at a normal load of 200 mN.

Different from the work by Yang et al. [15], we are focused on the local deformation behavior of the center portion of the transverse cross-section of the cold-rolled specimens. Figure 5 shows the load–displacement curves for the indentations of the as-annealed and cold-rolled AA6061 specimens with the maximum normal load of 200 mN, which exhibits a similar trend to the loading–unloading curves for the normal load of 400 mN, as reported by Zhou et al. [20]. There are no pop-in and pop-out events for the loading and unloading phases. Both the maximum indentation depth and the contact depth are decreasing functions of the thickness reduction, revealing the increase in the resistance to the penetration of the indenter onto the Al alloy with increasing the plastic deformation through the cold rolling. Such behavior is attributed to the rolling-induced increase of the dislocation density in the plastically deformed Al alloy. This trend is similar to the trend that the volume fraction of the recrystallization textures is a decreasing function of Δt/t_0_, as shown in Figure 4. With the increase of the cold rolling, the number of grains with less internal stresses (dislocation density) decreases, and there are more and more grains with large internal stresses (dislocation density) in the Al alloy. 

**Figure 6:**
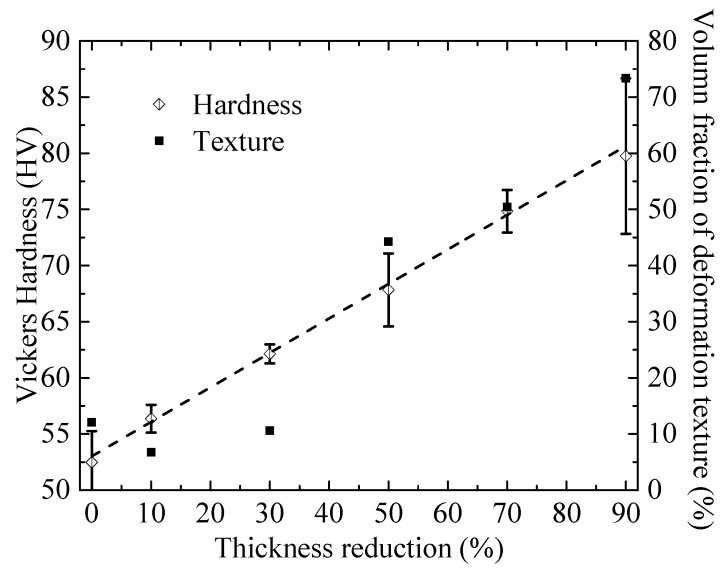
Dependence of the Vickers hardness on the thickness reduction for the indentation of the as-annealed and cold-rolled specimens at the normal load of 200 mN.

The Vickers hardness, *H_V_*, which represents the resistance to local plastic deformation, can be calculated as:

(1) $$H_{V}=\;\frac{1.854F}{D^{2}},$$

where *F* is the normal load, and *D* is the diagonal length of the impression on the indented surface. Figure 6 shows the dependence of the Vickers hardness on the thickness reduction. The Vickers hardness is an approximately linearly increasing function of the thickness reduction, as expected, due to the increase of the dislocation density in the plastically deformed AA6061 specimens. 

From Figure 4, we note that the volume fractions of the deformation texture also increase with the thickness reduction. To examine if there exists any connection between the change of the Vickers hardness and the deformation texture, the volume fraction of the deformation texture is also included in Figure 6. It is interesting to note that the volume fraction of the deformation texture displays almost the same increasing trend as the Vickers hardness for Δt/t_0_ > 30%. For Δt/t_0_ ≤ 30%, the trends are different. Such behavior may imply the possible connection between the deformation texture and the dislocation density, i.e., the variation of the deformation texture (Copper, S and Brass components) may be associated with the increase of the dislocation density (the rate of work hardening) and the dislocation motion in FCC (face-centered cubic) metals with severe deformation. 

It is known that there exists strain hardening associated with multiplication of dislocations and the increase of dislocation density, which leads to the increase in the resistance to the dislocation motion. Generally, the strain dependence of plastically deformed metal, σ, can be expressed as [21]

(2) $$\sigma =\sigma_{0}+\alpha \mu b\varepsilon_{eff}^{n},$$

where σ_0_ is the flow stress at the reference state of ε*_eff_* = 0, ε*_eff_* is the effective strain, α is a constant, μ is the shear modulus, *n* is strain exponent, and *b* is the magnitude of Burgers vector. For a cold-rolled AA6061 Al sheet, the effective strain can be calculated from the thickness reduction as:

(3) $$\varepsilon_{eff}=\ln(\frac{t_{0}}{t})=-\ln(1-\frac{\Delta t}{t_{0}}),$$

with *t* being the thickness of the cold-rolled AA6061 Al sheet, *t*_0_ being the thickness at the state of ε*_eff_* = 0, and ∆*t*/*t*_0_ being the thickness reduction. Substituting Equation (3) into (2) and using the relationship of *H_V_ =* 3σ, we obtain:

(4) $$\begin{array}{l} H_{V}=H_{0}+3\alpha \mu b\varepsilon_{eff}^{n}=H_{0}+3\alpha \mu b{\left[-\ln(1-\frac{\Delta t}{t_{0}})\right]}^{n} \end{array}$$

**Figure 7:**
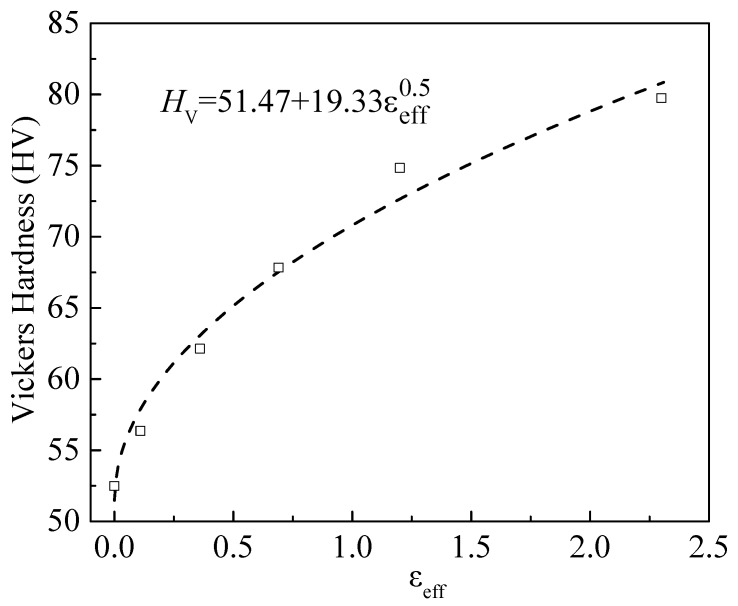
Dependence of Vickers hardness on effective strain for the as-annealed and cold-rolled specimens with the indentations performed at the normal load of 200 mN.

Figure 7 depicts the dependence of the Vickers hardness on the effective strain for the as-annealed and cold-rolled specimens with the indentations performed at the normal load of 200 mN. Using Equation (4), we fit the data shown in Figure 7, and include the fitting curve in Figure 7. Evidently, Equation (4) fits the data well, and the fitting result yields *n* = 0.5. This result confirms the dominant role of the dislocation motion in controlling the local deformation of the cold-rolled AA6061 alloy. Such a result together with the trend shown in Figure 6 indirectly reveals the effect of dislocation motion on the evolution of the deformation texture and supports that the variation of the deformation texture can be attributed to the increase of the dislocation density and the dislocation motion.

It is known that the plastic energy dissipated during mechanical deformation is associated with the ductility of the material. Using the loading–unloading curves shown in Figure 5, one can calculate the plastic energy dissipated in an indentation cycle, *E_plastic_*, as:

(5) $$E_{plastic}=\int_{0}^{\delta_{\max}}Fd\delta -\int_{\delta_{r}}^{\delta_{\max}}F_{un}d\delta ,$$

where δ_max_ is the maximum indentation depth, δ*_r_*is the residual indentation depth, and *F_un_* is the normal load applied to the indenter for the unloading process. 

**Figure 8:**
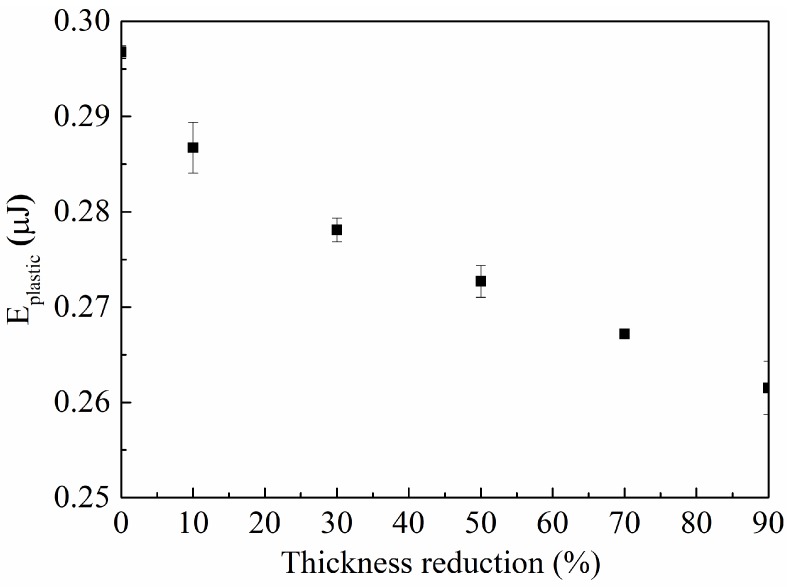
Plastic energy dissipated in the indentation of the as-annealed and cold-rolled specimens at the normal load of 200 mN.

Figure 8 shows the variation of the plastic energy dissipated in an indentation cycle for indentations of the as-annealed and cold-rolled specimens at the normal load of 200 mN. It is evident that *E_plastic_* is a nonlinear decreasing function of Δt/t_0_. The specimen with Δt/t_0_ = 0 has the largest energy dissipation and ductility, and the cold-rolled specimen of 90% in the thickness reduction has the least energy dissipation and ductility. Such a result is due to that plastic deformation causes the increase in dislocation density. The larger the thickness reduction, the more severe is the plastic deformation, and the higher is the dislocation density. Note that plastic deformation is a function of the microstructure (internal stress) in the materials, which needs to be considered in the calculation of the energy dissipation for the plastic deformation of crystallized metals.

## 4. Conclusions

AA6xxx Al alloys have potential as structural materials in the applications of automobile and aerospace industries. It is of great importance to investigate the effect of plastic deformation on the microstructure and mechanical behavior of AA6xxx Al alloys for analyzing the structural integrity of the mechanical structures made from AA6xxx Al alloys. 

Using the cold-rolling processing, plastic deformation was introduced in AA6061 Al sheets. Grains elongate along the rolling direction during the cold rolling, which causes the changes of textures. The grain elongation is an increase function of Δt/t_0_ likely due to the compression deformation of grains during the rolling. A slight difference in the grain sizes exist between the material near the free surface and the material around the center portion for a small reduction in the thickness. Increasing the thickness reduction (plastic deformation) leads to the decrease of the volume fraction of the recrystallization texture and the Cube component as well as the increase of the volume fraction of the deformation texture due to the transform of the Cube component and random texture to the components of the deformation texture. 

Microindentations of the as-annealed and cold-rolled AA6061 Al sheets were performed. The Vickers hardness is an approximately linearly increasing function of the thickness reduction, and displays almost the same increase trend as the volume fraction of the deformation texture for the thickness reduction larger than 30%. Such behavior may imply that the variation of the deformation texture is dependent on the dislocation density and the dislocation motion in FCC metals with severe deformation. A simple relation between the Vickers hardness and the thickness reduction is established and used to curve-fit the experimental results. The curve fitting gives the strain exponent of 0.5, which confirms the dominant role of the dislocation motion in controlling the local deformation of the cold-rolled AA6061 alloy.

## Figures and Tables

**Table 1:** Composition of AA6061 Al alloy (wt.%).

Sample	Mg	Si	Fe	Cu	Cr	Al
AA6061	1.0	0.6	0.34	0.27	0.2	Bal.
